# Potential mechanisms of cancer stem‐like progenitor T‐cell bio‐behaviours

**DOI:** 10.1002/ctm2.1817

**Published:** 2024-08-21

**Authors:** Ling Ni

**Affiliations:** ^1^ Institute for Immunology and School of Basic Medicine Tsinghua University Beijing China

**Keywords:** cancer, CD8^+^ T cells, immune checkpoint inhibitors, Tex‐int cells, Tex‐term cells, Tpex cells

## Abstract

In situations involving continuous exposure to antigens, such as chronic infections or cancer, antigen‐specific CD8^+^ T cells can become dysfunctional or exhausted. This change is marked by increased expression levels of inhibitory receptors (PD‐1 and Tim‐3). Stem‐like progenitor exhausted (Tpex) cells, a subset of exhausted cells that express TCF‐1 and are mainly found in the lymph nodes, demonstrate the ability to self‐renew and exhibit a high rate of proliferation. Tpex cells can further differentiate into transitional intermediate exhausted (Tex‐int) cells and terminally exhausted (Tex‐term) cells. Alternatively, they can directly differentiate into Tex‐term cells. Tpex cells are the predominant subset that respond to immune checkpoint inhibitors (ICI), making them a prime candidate for improving the efficacy of ICI therapy. This review article aimed to present the latest developments in the field of Tpex formation, expansion, and differentiation in the context of cancer, as well as their responses to ICIs in cancer immunotherapy. Consequently, it may be possible to develop novel treatments that exclusively target Tpex cells, thus improving overall treatment outcomes.

**Key points:**

- Tpex cells are located in lymph nodes and TLS.
- Several pathways control the differentiation trajectories of Tpex cells, including epigenetic factors, transcription factors, cytokines, age, sex, etc.

## Graphical abstract

(1) Several pathways involving epigenetic factors, such as cBAF, control the differentiation trajectories of Tpex cells. (2) Bio‐behaviour of cancer stem‐like progenitor T cells is regulated by transcription factors such as IKAROS. (3) The cancer stem‐like progenitor T‐cell bio‐behaviour is regulated by cytokines such as IL‐2. (4) Other factors, such as MAP4K1, regulate the cancer stem‐like progenitor T‐cell bio‐behaviour

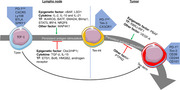

## 1 INTRODUCTION

Cancer immunotherapy aims to harness the immune system to control cancer cells.^1^ This approach offers a more tailored and less toxic treatment option that has been shown to promote durable responses and improve outcomes in some cancer patients. Multiple immunotherapies, such as chimeric antigen receptor (CAR)‐T cells, checkpoint inhibitors, cancer vaccines, and tumour‐infiltrating lymphocytes (TILs), all aim to promote CD8^+^ T‐cell responses.^2^ This is achieved by activating, amplifying, or redirecting T cells to recognize and eliminate cancer cells, thereby providing a targeted and effective approach to cancer treatment.

In the context of persistent exposure to antigens, such as chronic infection or cancer, antigen‐specific CD8^+^ T cells undergo exhaustion or dysfunction.^3^ This state is characterized by increased expression levels of inhibitory receptors, such as PD‐1. One subset of exhausted cells, designated as stem‐like progenitor exhausted (Tpex) cells, express TCF‐1, allowing them to self‐renew with a high rate of proliferation. Tpex cells can either continue to differentiate into transitional intermediate exhausted (Tex‐int) cells and finally into terminally exhausted (Tex‐term) cells,^4^, ^5^, ^6^, ^7^, ^8^, ^9^ or can differentiate directly into Tex‐term cells.^10^ However, during this differentiation process, they lose both their proliferative capacity and effector functions.

Tpex cells play a pivotal role in the maintenance of antigen‐specific CD8^+^ T‐cell responses. Following anti‐PD‐1/PD‐L1 immune checkpoint blockade (ICB) treatment, Tpex cells represent the predominant subset of exhausted cells that undergo expansion and differentiation into more differentiated subsets.^6^ This expansion contributes to more Tex‐term cells in the tumour. Interestingly, Tex‐term cells themselves fail to exhibit substantial proliferation in response to ICB treatment, although they may become more activated. Moreover, Tex‐term cells express the highest levels of PD‐1 compared with Tpex and Tex‐int cells. Prolonged survival following anti‐PD‐1 treatment in melanoma patients is correlated with an increased frequency of TCF‐1^+^ CD8^+^ TILs compared with TCF‐1^−^ CD8^+^ TILs.^11^ Tpex cells are a prime candidate for improving the efficacy of immune checkpoint inhibitor (ICI) therapy.

This review paper presents a summary of recent advances in our understanding of the mechanisms underlying the formation, expansion, and differentiation of Tpex in cancer. These insights may inform the development of targeted therapies aimed at enhancing the survival and functionality of Tpex cells, potentially improving overall treatment outcomes.

## 2 THE LOCALIZATION OF TPEX CELLS

**FIGURE 1:**
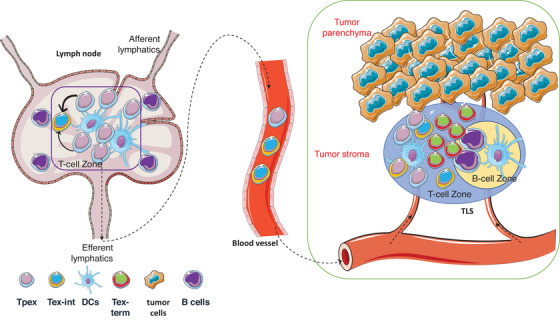
Tpex cells are located in the lymph nodes and TLS. Most Tpex cells are sustained and activated in the tumour‐draining lymph nodes, serving as progenitors for their more differentiated counterparts within the tumour. Some Tpex cells migrate into tumours via blood vessels and are found in the TLS of the tumour stroma, which supports the quiescence and maintenance of Tpex cells within the tumours. TLS, tertiary lymphoid structure; Tpex, stem‐like progenitor exhausted; Tex‐int, transitional intermediate exhausted; Tex‐term, terminally exhausted.

In mice with autochthonous lung cancer, the tumour‐draining lymph node (tdLN) has been identified as the predominant site where Tpex cells are sustained and stimulated. Connolly et al.^12^ observed a limited presence of Tpex cells in lung tumours throughout progression. In contrast, a significant proportion of Tpex cells remained localized in tdLNs, and this population remained constant in number despite alterations in the tumour microenvironment (TME). Remarkably, the T cells in tdLNs were identified as precursors of their more differentiated counterparts in the tumour, and they exhibited clonal relatedness.^12^ The Tpex reservoir in the tdLNs played a crucial role in sustaining anti‐tumour T cells during tumour development and served as a protective mechanism against the terminal differentiation that occurs in the TME. In another study using an autochthonous model of lung adenocarcinoma, Schenkel et al.^13^ also found that the frequency of Tpex cells in the tdLNs remained stable. When the egress from the tdLN was blocked, the frequency of intra‐tumoral Tpex cells was reduced. The number of conventional type I dendritic cells (cDC1) in the tdLN decreased during tumour growth, but treatment with Fms‐like tyrosine kinase‐3 ligand (Flt3L) plus anti‐CD40 led to cDC1 maturation, followed by the recovery of Tpex cell frequencies and a reduction in the tumour burden.^13^ This indicated that cDC1s in the tdLN serve to maintain a reservoir of Tpex cells (Figure 1), and their decline contributes to compromised anti‐tumour immunity.

In patients with lung adenocarcinoma, Connolly et al.^12^ observed that the majority of Tpex cells were present in non‐metastatic lung‐draining LNs, which was in line with the findings in mice, where Tpex cells were predominantly located in tdLNs. In addition, in patients with advanced head and neck squamous cell carcinoma treated with surgery and perioperative anti‐PD‐L1 immunotherapy (atezolizumab), there was a notable presence of Tpex in regional, uninvolved LNs that shared clonal relationships with Tex‐term cells in the tumour.^14^ After anti‐PD‐L1 ICB, the percentage of Tpex cells in uninvolved LNs decreased, but they were observed to localize near DCs and differentiate into Tex‐int cells.^14^ This observed localization was consistent with a process of activation and differentiation, suggesting that anti‐PD‐L1 immunotherapy induced changes in the Tpex cell population in uninvolved LNs. Thus, Tpex cells in cancer patients were also mainly located in the LN.

A proportion of the Tpex cells that migrated into the tumours were observed to localize in tertiary lymphoid structures (TLSs) (Figure 1). In the context of patients with stage I–IV non‐small‐cell lung cancer (NSCLC), Tpex cells were observed to be located in the TLSs rather than in the tumour parenchyma.^15^ These Tpex cells were found in the TLSs both within and at the tumour periphery, as well as in the TLSs close to the tumour parenchyma. These observations indicated that TLSs act as a protective microenvironment, facilitating the maintenance of Tpex cells in tumours. In primary prostate cancer, Calagua et al.^16^ demonstrated that most PD‐1^+^ CD8^+^ T cells in the TME failed to express Tex‐term exhaustion markers (Tim‐3 or LAG3), whereas a subset expressed TCF‐1, indicating a progenitor function. Nevertheless, these cells were observed in antigen‐presenting cell (APC) niches near MHC II^+^ cells.

## 3 TPEX CELL EXPANSION AND DIFFERENTIATION

The mechanism underlying the capacity of critical lymphocytes to continuously produce the progeny of differentiated cells remains poorly understood. Wang et al.^17^ demonstrated that a dividing Tpex cell exhibits inhibitory and activating signalling components at its spindle on two opposite ends, thereby generating a daughter cell destined for functional activity, while the sister cell could undergo the same process. In their study, the researchers employed three‐dimensional microscopy in a melanoma murine model to identify that an activating hub, comprising polarized CD28, CD3, and PI3K activity, occurred at the presumed immunological synapse. In contrast, the inhibitory hub with polarized CD73 and PD‐1 was located at the opposite pole of the mitotic blasts.^17^ Tpex cells from ICB‐treated and control mice led to the differentiation of a TCF‐1^−^ daughter cell with an inherited PI3K activation hub and a self‐renewing TCF‐1^+^ sister cell with a discordant fate. The dynamic organization of opposing inhibitory and activating signalling poles that occurred during the mitotic lymphocytes’ process may explain the durability of specific immunity.^17^

**FIGURE 2:**
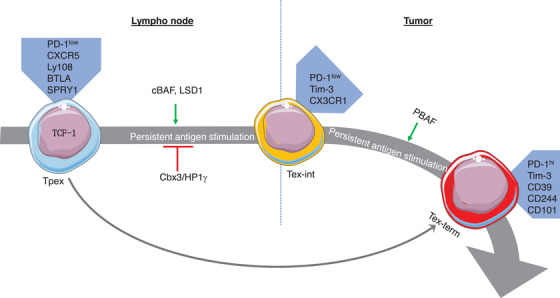
The mechanisms controlling the differentiation of exhausted T‐cell subsets with epigenetic factors. TCF‐1^+^ Tpex cells expressing surface markers such as PD‐1, CXCR5, Ly108, BTLA, and SPRY1 demonstrate self‐renewal ability and have a high rate of proliferation. These Tpex cells can either differentiate into Tex‐int cells with surface expression of PD‐1, Tim‐3, and CX3CR, and subsequently into Tex‐term cells, or directly into Tex‐term cells with surface markers of PD‐1, Tim‐3, CD39, CD244, and CD101. Several pathways involving epigenetic factors control the differentiation trajectories of Tpex cells. Tpex, stem‐like progenitor exhausted; Tex‐int, transitional intermediate exhausted; Tex‐term, terminally exhausted.

**FIGURE 3:**
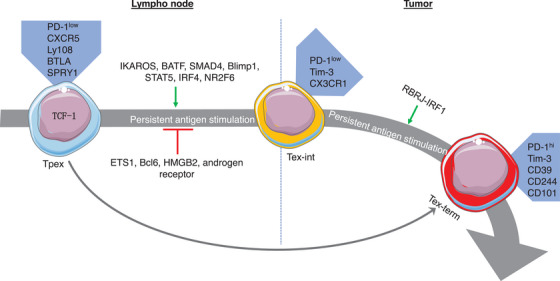
Bio‐behaviour of cancer stem‐like progenitor T cells with transcription factors. TCF‐1^+^ Tpex cells, which have the ability to self‐renew with a high proliferation rate, can differentiate either into Tex‐int cells or directly into Tex‐term cells. The mechanisms by which transcription factors control the differentiation of exhausted T‐cell subsets have been studied. Tpex, stem‐like progenitor exhausted; Tex‐int, transitional intermediate exhausted; Tex‐term, terminally exhausted.

**FIGURE 4:**
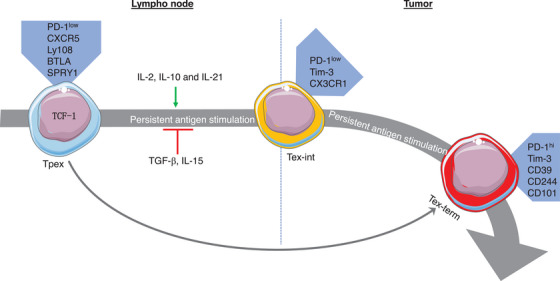
The mechanisms of cancer stem‐like progenitor T‐cell bio‐behaviour involving cytokines. TCF‐1+ Tpex cells demonstrating self‐renewal ability with a high rate of proliferation can either differentiate into Tex‐int cells or directly into Tex‐term cells. The mechanisms that control the differentiation of exhausted T‐cell subsets by means of cytokines have been studied. Tpex, stem‐like progenitor exhausted; Tex‐int, transitional intermediate exhausted; Tex‐term, terminally exhausted.

**FIGURE 5:**
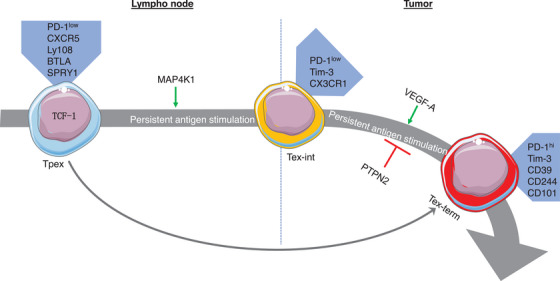
Bio‐behaviour of cancer stem‐like progenitor T cells with other factors. The mechanisms controlling the differentiation of exhausted T‐cell subsets by other factors have been investigated. Tpex, stem‐like progenitor exhausted; Tex‐int, transitional intermediate exhausted; Tex‐term, terminally exhausted.

Beltra et al.^8^ identified two TCF‐1^+^ progenitor subsets; one was quiescent and tissue‐restricted, and the other was more accessible in the blood and slowly lost TCF‐1 as it divided to become a Tex‐int subset. This Tex‐int subset demonstrated effector function and increased following anti‐PD‐L1 ICB, but eventually became the Tex‐term subset. The mechanisms controlling the subset transitions were defined using transcriptional and epigenetic analyses, which revealed a significant interplay between TCF‐1, Tox, and T‐bet in the process.^8^ Several pathways that control the differentiation trajectories of Tpex cells have been studied, including changes in chromatin structure (Figure 2). Regulation of transcription factors (Figure 3), cytokines (Figure 4), and other factors (Figure 5) has also been implicated in this differentiation process.

### 3.1 Epigenetic factors

Two variants of the SWI/SNF chromatin remodelling complex have been implicated in the differentiation of Tpex cells. Depletion of the canonical SWI/SNF form, polybromo‐associated BRG1/BRM‐associated factor (PBAF), was observed to enhance the proliferation and survival of exhausted CD8^+^ T cells.^18^ Mechanistically, PBAF was found to regulate the transcriptional and epigenetic transition from Tpex cells to more differentiated TCF‐1^−^ Tex subsets. In contrast, canonical BAF (cBAF), another variant of the SWI/SNF complex, was found to be essential for the induction of effector‐like exhausted T cells. This suggests that the balance between PBAF and cBAF coordinates the differentiation of exhausted T subsets (Figure 2).^18^

Targeting PBAF alone or in combination with anti‐PD‐L1 ICB improved tumour control, indicating that PBAF could be a promising therapeutic candidate in cancer immunotherapy. In their study, Khavel et al.^19^ utilized B16‐F10 melanoma tumour cells producing LCMV GP33‐41 peptide epitope (termed B16‐GP33). The transfer of *Arid2* (encoding PBAF)‐deleted CD8^+^ P14 T cells resulted in significantly lower tumour burdens compared with the control group. Moreover, in both the tumour and tdLN, there were significantly more *Arid2*‐deficient CD8^+^ P14 cells. Notably, P14 cells lacking *Arid2* decreased PD‐1 and LAG3 expression, accompanied by a marked increase in CXCR6 expression.^19^ Therefore, the findings suggested that the absence of *Arid2* results in the generation of highly proliferative and less exhausted intra‐tumoral effector CD8^+^ T cells, ultimately leading to enhanced tumour control. To assess the CD8^+^ T‐cell response independently of tumour volume, B16‐GP33‐bearing recipient mice were treated with equal numbers of control and sgArid CD8^+^ P14 T cells.^19^ In the same TME, *Arid2* deficiency conferred enhanced proliferative ability and decreased terminal exhaustion in tumour‐specific CD8^+^ T cells compared with their wild‐type control. Notably, no obvious differences in granzyme B expression between the group with Arid2‐deficient CD8^+^ T cells and the control group were detected.^19^ Overall, these findings demonstrated that the PBAF complex plays a crucial role in promoting exhaustion in tumour antigen‐specific CD8^+^ T cells. The disruption of PBAF complex activity in CD8^+^ T cells resulted in increased proliferative ability and decreased exhaustion, rendering the PBAF an appealing target for cancer immunotherapy.

Multiple components of the mammalian cBAF have been identified as negative regulators of memory T‐cell generation. While multiple cBAF complex components are required for the differentiation of activated CD8^+^ T cells into effector T cells, their deficiency promotes the formation of memory T cells in vivo. During the initial division of activated CD8^+^ T cells, MYC and cBAF were shown to often co‐assemble to the two daughter cells asymmetrically. Daughter cells that expressed high MYC and cBAF were more likely to follow an effector T‐cell fate trajectory, whereas those with low cBAF and MYC were more prone to differentiate into memory T cells. The cBAF complex and MYC cooperatively established the chromatin landscape in activated CD8^+^ T cells. In particular, during the first 48 h of activation, the use of a putative cBAF inhibitor demonstrated enhanced efficacy in a mouse tumour model. The findings indicated that cBAF can be manipulated early in T‐cell differentiation to enhance cancer immunotherapy and positively impact T‐cell memory.^20^

In addition to the SWI/SNF complex, histone demethylase LSD1 also plays a role in regulating the differentiation of Tpex cells. Liu et al.^21^ discovered that LSD1 enforced an epigenetic program in Tpex cells, antagonizing the TCF‐1‐mediated maintenance of the progenitor status and promoting terminal differentiation (Figure 2). As a result, genetic perturbation or the use of small molecules that target LSD1 resulted in an enhanced persistence of Tpex cells. This persistence provided a continuous source for proliferative differentiation into a great number of Tex‐term cells with potent tumour‐killing cytotoxicity, ultimately resulting in durable and effective responses to anti‐PD‐1 ICB.^21^ Taken together, these findings collectively offer crucial insights into the epigenetic mechanisms governing T‐cell exhaustion.

Cbx3/HP1γ is a histone reader involved in the control of Tpex cell differentiation. Targeting Cbx3/HP1γ in CD8^+^ T cells has been demonstrated to improve chromatin remodelling and transcription initiation (Figure 2), resulting in enhanced transcriptional activity at Lef1 and Il21r.^22^ IL‐21R and LEF‐1 have been shown to play a crucial role in the control of ovarian cancer, melanoma, and neuroblastoma using CD8^+^ effector T cells lacking Cbx3/HP1γ in preclinical models. The remodelling of the tumour chemokine/receptor landscape and the increased persistence of CD8^+^ T cells lacking Cbx3/HP1γ allows their optimum invasion into tumours at the cost of CD4^+^ Tregs. The enhancement of CD8^+^ T‐cell effector function through Cbx3/HP1γ deficiency is a promising approach for T cell‐based therapy targeting ICB‐resistant and non‐responsive solid tumours.^22^ This suggests that targeting Cbx3/HP1γ is a viable option for the treatment of resistant and nonresponsive solid tumours.

### 3.2 Cytokines and transcription factors

When analyzing transcription factors that regulate T‐cell exhaustion among differentially expressed genes in the CD8^+^ TILs of human NSCLC and melanoma, TOX was observed as the only transcription factor expressed in both tumour types.^23^ Higher levels of TOX were observed as CD8^+^ T cells became more exhausted, indicating an association between the expression of TOX and the severity of tumour‐infiltrating T‐cell exhaustion. Knockdown experiments revealed that TOX promotes T‐cell exhaustion by upregulating inhibitory molecules in cancer cells, leading to an increase in the expression of PD‐1, CTLA‐4, Tim‐3, and TIGIT, while inhibition of TOX has been found to reduce T‐cell exhaustion and improve ICB efficacy,^23^ indicating that TOX stimulates Tpex cells to differentiate into Tex‐term cells.

In their study, Chi and colleagues employed single‐cell CRISPR screens in vivo to systematically map gene regulatory networks and identify checkpoints for Tpex cell differentiation.^24^ Their findings revealed that the exit from Tpex cell quiescence triggered successive differentiation into Tex‐int cells, which were differentially regulated by IKAROS and ETS1. Deficiencies in IKAROS and ETS1 resulted in decreased and increased mTORC1‐associated metabolic activities, respectively. After receiving ICB, the cells lacking IKAROS were found to be Tpex cells with limited differentiation potential. On the other hand, an enhanced anti‐tumour immune response and ICB efficacy were observed when differentiation of Tpex into Tex‐int cells was increased and metabolic rewiring was achieved via targeting ETS1. Mechanistically, it has been identified that TCF‐1 and basic leucine zipper ATF‐Like transcription factor (BATF) represent the respective targets for IKAROS and ETS1. Furthermore, another axis called RBPJ–IRF1 has been shown to promote the differentiation of Tex‐int to Tex‐term cells. By targeting RBPJ, it was found to be possible to improve the reprogramming of Tex cells towards the proliferative state, which in turn led to better therapeutic effects and efficacy of ICB.^24^ Overall, these findings indicated that the differentiation of Tpex cells into proliferative Tex cells is a key determinant of anti‐tumour effects. This provides a comprehensive understanding of the cell fate regulome and reprogrammable determinants in cancer immunity (Figure 3).

In patients with metastatic cancers, TGF‐β signalling is linked to a lack of response to ICB, particularly among those exhibiting the immune‐excluded phenotype (Figure 4). The transition of tumours from an excluded to an inflamed phenotype requires the inhibition of PD‐L1 and TGF‐β signalling. In an EMT6 tumour model in female mice, Castiglioni et al.^25^ demonstrated that TGF‐β and PD‐L1 jointly restricted the expansion of intra‐tumoral Tpex cells and hindered the replacement of Tpex cells with non‐exhausted T effector cells. Upon exposure to a combined blockade of TGF‐β and PD‐L1, IFN‐γ (high) CD8^+^ T effector cells exhibited enhanced motility and accumulated in the tumour. It was of note that the efficacy of anti‐PD‐L1/anti‐TGF‐β therapy was eliminated by the blockade of IFN‐γ. These findings suggested that TGF‐β, in conjunction with PD‐L1, prevents the expansion of Tpex cells, thereby maintaining the T‐cell compartment in an exhausted state.^25^

Mechanistically, the TGF‐β‐induced restraint of Tpex cell expansion is mediated by the transcription factor Bcl6. Sun et al.^26^ revealed that TGF‐β‐SMAD2 signaling upregulated BCL6 expression by CD8^+^ T cells. BCL6, in turn, inhibited the generation of tumour‐specific Tex‐term cells from Tpex cells. Bcl6 deficiency led to a reduction in the persistence of Tpex cells, with no impact on their generation.^26^ Disruption of long‐term tumour control was observed. Tumour‐specific T cells in tdLNs express high levels of BCL6, which is correlated with T‐cell exhaustion, particularly TOX^+^ TCF‐1^+^ Tpex cells in both LNs and tumours. BLIMP1 antagonizes the expression and function of BCL6 during the differentiation of Tfh cells. The ChIP‐seq data for BCL6 indicated its direct occupancy at several sites in the Prdm1 gene (encoding BLIMP1) within CD8^+^ T cells. These sites were distributed across intron and distal regulatory regions.^26^ Similarly, BLIMP1 was directly bound to the Bcl6 locus, including both the gene itself and various distal intergenic loci in CD8^+^ T cells. Consequently, Prdm1 knockout enhanced the Tpex cell program and significantly enhanced the efficacy of anti‐PD‐1 ICB. The BLIMP1 antagonistic pathway plays a crucial role in regulating anti‐tumour T cells, offering potential benefits for the development of enduring and effective cancer immunotherapy.^26^

SMAD4, a vital component of the TGF‐β signalling pathway, is instrumental in promoting the effector function of CD8^+^ T cells in both tumour and infection models.^27^ SMAD4 plays a role in regulating the transcriptional activity of CD8^+^ T cells including their activation and cytotoxicity. This regulation relies on the T‐cell receptor (TCR) rather than the TGF‐β pathway. After TCR activation, SMAD4 was observed to be translocated to the nucleus, thereby stimulating the expression of genes that encode cytotoxic molecules and TCR signalling components in CD8^+^ T cells. This finding reinforced the T‐cell effector function.^27^

Common gamma chain cytokines, namely IL‐15, IL‐2, and IL‐21, play a role in Tpex cell differentiation (Figure 4). IL‐2 signalling was observed to inhibit various Tpex‐associated gene expressions (Tcf7, Id3, Slamf6) while upregulating Tex‐related gene expressions (Prdm1, Id2, Havcr2).^26^ Mechanistically, in vitro‐activated CD8^+^ T cells demonstrated a significant interaction between IL‐2‐stimulated‐pSTAT5 and the Bcl6 promoter, influencing Bcl6 transcription and inhibiting Tpex differentiation. Moreover, persistent IL‐2 stimulation, in the absence of Prdm1, failed to induce CD25^high^ Tim‐3^+^ CD8^+^ T cells and reduce TCF‐1 expression. This suggested that persistent IL‐2 treatment effectively induced the generation of Tim‐3^+^ CD8^+^ T cells in vitro, dependent on BLIMP1.^26^ The results implied that the IL‐2‐STAT5 signalling pathway inhibited Bcl6 transcription and promoted Blimp1 transcription, potentially through direct binding of STAT5 to the Bcl6 promoter. This, in turn, impeded Tpex differentiation.^26^

IL‐21 played a similar role to IL‐2 in the Tpex cell differentiation process, as evidenced by the findings of Topchyan et al.^28^ Their study identified BATF as a crucial factor in the maintenance of CD8^+^ T‐cell effector function in tumours.^28^ This was demonstrated to occur downstream of IL‐21 signalling. The deletion of BATF in CD8^+^ T cells impaired tumour clearance, while BATF overexpression promoted the effector function, improving tumour control independently of CD4^+^ helper T cells. Transcriptomic analyses revealed increased expression of costimulatory receptors, transcriptional regulators, and effector molecules in BATF‐overexpressing CD8^+^ T cells. This unveiled an IL‐21–BATF axis derived from CD4^+^ T cells, offering therapeutic insights into enhancing CD8^+^ T‐cell effector function against cancer. Additionally, Sun et al.^29^ found that IL‐21‐induced pSTAT3 is crucial for terminally differentiated effector CD8^+^ T cells in the TME. IL‐10 and IL‐21, but not IL‐6, can activate STAT3, which promotes effector function‐associated genes while suppressing those expressed by Tpex cells. STAT3 collaborates with IRF4 and BATF to induce chromatin activation at the loci of effector genes. In contrast, Lee et al.^30^ investigated how CD8^+^ TILs from patients with renal cell carcinoma responded to IL‐15. Ex vivo IL‐15 treatment significantly increased Tpex cell expansion within PD‐1^+^ CD8^+^ TILs compared with Tex cells, suggesting that IL‐15 may enhance Tpex cell self‐renewal, with therapeutic implications.

The nuclear receptor (NR) NR2F6 was identified as a regulator of anti‐tumour immunity.^31^ It was isolated from a set of 48 candidate NRs according to its expression pattern in melanoma patient samples. In a mouse melanoma model, the loss of NR2F6 enhanced the response to anti‐PD‐1 ICB. The knockout of NR2F6 in both B16F10 and YUMM1.7 melanoma cells resulted in a reduction in tumour growth in immune‐competent mice, but not in those lacking immune competence.^31^ This indicated that NR2F6 promoted the generation of Tex‐term cells from Tpex cells. Furthermore, the inoculation of NR2F6 knockout mice with NR2F6 knockdown melanoma cells resulted in a further decrease in tumour progression compared with NR2F6 wild‐type mice. The intrinsic and extrinsic roles of NR2F6 in tumours justify its consideration in the development of effective anticancer therapies.^31^

The expression of the high mobility group box 2 (HMGB2) protein was increased and prolonged in exhausted CD8^+^ T cells.^32^ HMGB2 has been identified as a regulator responsible for the generation and maintenance of Tpex cells in tumours in a cell‐intrinsic manner. Despite the expression of TCF‐1 and TOX, essential master regulators, in Hmgb2^−/−^ CD8^+^ T cells, they failed to maintain Tpex differentiation and long‐term survival with persistent antigens.^32^ These findings highlighted HMGB2 as a critical regulator of CD8^+^ T cells, suggesting it could be a significant target for T cell‐based immunotherapies.

In general, many non‐reproductive organ cancers are more deadly in men than in women. Intra‐tumoural antigen‐specific Tcf7/TCF‐1^+^ Tpex cells were found to be present in greater numbers in males due to intrinsic androgen receptor (AR) function.^33^ The AR is a direct regulator of Tcf7, and its signalling induces the sex‐biased generation of the Tpex cell subset with impaired tumour control. The AR‐mediated predisposition to CD8^+^ T‐cell exhaustion results in reduced elimination of newly formed immunogenic malignant cells and contributes to a male bias in both tumour incidence and mortality, mainly because the androgen–AR axis inhibits the generation of tumour‐specific Tex‐term cells from Tpex cells. Thus, inhibition of the androgen–AR axis increased effector T‐cell differentiation and increased the efficacy of anti‐PD‐1 ICB.^33^ These findings illustrated the role of androgens in promoting CD8^+^ T‐cell exhaustion in cancer and highlighted opportunities for therapeutic development through understanding sex differences. In glioblastoma, male mice showed accelerated tumour growth.^34^ Moreover, a higher percentage of Tpex cells was observed in males that showed a better response to anti‐PD‐1 treatment. Lee et al.^34^ tested whether X chromosome inactivation escape genes were responsible for sex‐biased Tpex cell formation and found that female in vitro exhausted T cells expressed increased levels of *Kdm6a* compared with male T cells. Blockade of UTX (encoded by *Kdm6a*) resulted in a decrease in cytokine production (IFN‐γ) and an increase in exhaustion markers (PD‐1, TOX, and Tim‐3). Thus, female T cells are more likely to remain more functional and protect the host from cancer progression, in part, due to the protective capacity of higher levels of UTX. These findings suggested that gender‐specific approaches might enhance the therapeutic efficacy of immunotherapy in glioblastoma.

The transcription factor EGR2 is another regulator of Tpex differentiation. EGR2 expression in exhausted CD8^+^ T cells is driven by persistent antigen stimulation, leading to higher EGR2 expression compared with effector CD8^+^ T cells.^35^ Notably, EGR2 expression was found to be selective in the TCF‐1^+^ Tpex cells and was lost following the commitment to more differentiated states. Furthermore, EGR2 plays a critical role in regulating Tpex cell differentiation through both epigenetic and transcriptional regulation of the exhausted state. These findings highlighted the importance of EGR2 as a key epigenetic and transcriptional regulator of the exhausted state.^35^

### 3.3 Other factors

Tyrosine‐protein phosphatase non‐receptor type 2 (PTPN2) is a known regulator of Tpex cells that attenuates type 1 interferon signalling. Ptpn2‐deficient CD8^+^ T cells show an increase in the generation, proliferative potential, and cytotoxic activity of Tim‐3^+^ cells without alteration of Tpex cells, leading to better tumour control (Figure 5). Ptpn2 knockout in all hematopoietic cells leads to better tumour control and enhanced anti‐PD‐1 responses to B16 tumours. These results suggested that promoting the number of cytotoxic Tim‐3^+^ CD8^+^ T cells may be a promising strategy for effective anti‐tumour immune responses and that PTPN2 in immune cells may be a useful target for cancer immunotherapy.^36^ High expression of mitogen‐activated protein kinase kinase kinase kinase 1 (MAP4K1) correlated with the severity of T‐cell exhaustion and a reduction in patient survival across multiple cancer types.^37^ In MAP4K1^−/−^ mice, tumours grew more slowly, and intra‐tumoural T cells were less exhausted and more proliferative. In multiple mouse tumour models, pharmacological inhibition, genetic depletion, or PROTAC‐induced degradation of MAP4K1 were shown to be more effective than genetic depletion of PD‐1 in CAR‐T cells in improving the efficacy of CAR‐T cell‐based therapies.^37^ This study demonstrated that MAP4K1 is a regulator of T‐cell dysfunction and a potential target for enhancing anti‐tumour immune responses (Figure 5).

Neuropilin‐1 (NRP1) is an immune checkpoint that inhibits Tpex cell self‐renewal, and mice with NRP1‐deficient CD8^+^ T cells were protected against tumour rechallenge and showed an enhanced response to anti‐PD1 immunotherapy.^38^ NRP1 limited Tpex cell self‐renewal and reduced c‐Jun/AP‐1 expression following TCR restimulation. Blockade of NRP1 might promote the generation of long‐lived T cells specific to tumour antigens, which are crucial for sustained anti‐tumour immune responses.^38^ Despite unaltered primary tumour growth, NRP1 depletion may be a promising strategy for durable anti‐tumour immunity.

Maintenance of the redox balance may promote Tpex cell self‐renewal.^39^ Tpex cells rely on oxidative phosphorylation (OXPHOS) and mitochondrial fatty acid oxidation (FAO) for energy, while Tex‐term cells obtain energy by impaired glycolysis and OXPHOS.^40^ A bioenergetic compromise resulting from impaired ADP‐coupled OXPHOS limited nucleotide triphosphate synthesis and blocked T‐cell proliferation. Inhibition of mitochondrial OXPHOS suppressed proliferation and upregulated genes associated with T‐cell exhaustion while maintaining the redox balance during persistent antigen exposure promoted T‐cell self‐renewal and enhanced anti‐tumour immunity.^39^ Antioxidant treatment was found to improve the anti‐tumour responses of exhausted T cells.^39^ These findings suggested that OXPHOS plays a crucial role in T‐cell proliferation, as well as effector function and that maintaining the redox balance may promote T‐cell self‐renewal.

Bannoud et al.^41^ proposed that hypoxia, angiogenesis, and immunosuppression may be interrelated processes that drive tumour progression while hindering the efficacy of anti‐tumour therapies. They demonstrated that both hypoxia and vascular endothelial growth factor (VEGF)‐A, a regulated mediator of hypoxia, promote Tex‐term cell differentiation at the expense of Tpex cell subsets (Figure 5). This did not affect the expression of TNF‐α,  IFN‐γ, or granzyme B in the subsets. Notably, hypoxia enhanced the proangiogenic secretory profile of exhausted CD8^+^ T cells, with VEGF‐A being the primary factor produced by these cells under hypoxic conditions. These findings indicated that VEGF‐A is involved in the generation of Tex‐term cells during in vitro differentiation.^41^ Collectively, the results suggest that hypoxia, angiogenesis, and immunosuppression are mutually regulated, and provide a rationale for optimizing synergistic combinations of anti‐angiogenic and immunotherapeutic strategies to improve treatment efficacy. In contrast, ageing is recognized as a major risk factor for various types of cancers and may regulate the development of Tpex cells. A higher frequency of cytotoxic CD8^+^ T cells (CTLs) was observed in tumours from old mice compared with a higher percentage of exhausted CD8^+^ T cells in young mice. Moreover, the top 10 TCR clones in young mice were predominantly Tex‐term cells, whereas the top clones in old mice were predominantly CTLs. More Tpex cells and fewer ‘terminally’ exhausted phenotypes were observed in old mice than in young mice. Consistent with this, CD8^+^ T cells became more cytotoxic in old mice, while showing greater exhaustion levels in young mice. When CD8^+^ T cells were eliminated in old mice during tumour progression, the rate of tumour development significantly increased.^42^ Additionally, senescent features were only detected in exhausted CD8^+^ T cells, irrespective of the age of the mice. This finding suggested that the larger number of effector immune cells in old mice defended against tumour growth. These insights will help us to better understand the changes in tumour growth and the differential responsiveness of elderly patients to immunotherapeutic modulation.

## 4 TPEX CELL RESPONSE TO IMMUNE CHECKPOINT INHIBITORS

ICIs target Tpex cells and recover their effector function in cancer. In mouse tdLN, tumour‐specific Tpex cells were found to be closely linked with PD‐L1^+^ cDCs.^43^ PD‐L1 blockade therapy in the tdLN improved anti‐tumour immunity by transporting Tpex cells to the tumour site, leading to improved tumour control.^43^ Abundant PD‐1/PD‐L1 interactions were associated with early distant disease recurrence in patients with non‐metastatic melanoma in the tdLN, but not in the corresponding tumours,^43^ suggesting that PD‐L1 expression in the tdLN plays a critical role in orchestrating systemic anti‐tumour immune responses and identifying high‐risk patient populations suitable for anti‐PD‐1/PD‐L1 ICB.

In operable oesophagal squamous cell carcinoma (ESCC), Liu et al.^44^ analyzed tumours from ESCC patients undergoing neoadjuvant ICB treatment and found that a subset of exhausted CD8^+^ T cells expressed SPRY1, termed CD8^+^ Tex‐SPRY1. These cells displayed a Tpex phenotype that correlated with a complete response to ICB. Therefore, the CD8^+^ Tex‐SPRY1 cells can be regarded as an effective indicator of an improved response to and survival of ICB. Furthermore, they conducted independent studies in ICB and non‐ICB cohorts to demonstrate the ability of SPRY1 to enforce the Tpex phenotype and enhance ICB efficacy.^44^ These findings revealed the crucial role of Tpex‐like CD8^+^ Tex‐SPRY1 cells in effective responses to ICB in ESCC. This research signals the development of mechanistic biomarkers for personalized immunotherapy in the future.

Magen et al.^45^ found that DCs and Tfh cells play a role in regulating tumour‐specific Tpex cell differentiation following ICB. The ICB response was associated with intra‐tumoral clonal expansion of CXCL13^+^ CH25H^+^ IL‐21^+^ PD‐1^+^ CD4^+^ T helper cells (CXCL13^+^ Tfh) and effector‐like PD‐1^+^ Granzyme K^+^ CD8^+^ T cells, whereas non‐responders were predominantly characterized by Tex‐term cells. After treatment, CD4^+^ and CD8^+^ T‐cell clones which underwent expansion were present in pre‐treatment biopsies. Additionally, Tpex cells appeared to share clones mainly with Tex‐int cells in responders or Tex‐term cells in non‐responders, indicating local differentiation of CD8^+^ T cells in response to ICB.^45^ Notably, Tpex cells interacted with CXCL13^+^ Tfh in cellular triads surrounding maturation and regulatory molecule‐enriched DCs (mregDCs). Overall, these findings suggested that distinct intra‐tumoral niches, including mregDCs and CXCL13^+^ Tfh cells, regulated Tpex cell differentiation following ICB.

In a novel orthotopic hepatocellular carcinoma model, the few CD8^+^ TILs that were recovered were mainly Tex‐term TILs expressing high PD‐1.^46^ However, treatment with anti‐PD‐1/CTLA‐4 ICB resulted in more Tpex TILs, with very few Tex‐term cells being present in the tumours of the treated mice. It is noteworthy that naive tumour‐specific T cells transferred to untreated mice failed to expand in the tumours. However, they expanded significantly in response to treatment and generated Tpex TILs. Unexpectedly, the Tpex TILs demonstrated the capacity to mediate the anti‐tumour immune response following treatment, with minimal alterations observed in their transcriptional profile. Therefore, in this model, a few doses of ICIs administered during the priming phase of transferred tumour‐specific CD8^+^ T cells resulted in tumour clearance.^46^ Chen et al.^47^ also developed a potential approach to enhance the therapeutic efficacy of ICB by expanding intra‐tumoral Tpex cells. Their strategy involved the combination of an alarmin peptide called high‐mobility group nucleosome binding domain 1 (HMGN1) with PD‐L1 ICI. The immunostimulatory domain (EPKRR SARLS AKPPA KVEAK PKK) on HMGN1 was identified as a therapeutic peptide (minP1). The researchers tested the combined treatment of minP1 and PD‐L1 blockade on mice bearing B16F10, LLC, Colon26, or EO771 tumours, and observed durable tumour regression.^47^ The administration of MinP1 led to an increase in the number of mregDCs and a concomitant enhancement of their antigen‐presenting program. Furthermore, the combined therapy showed a synergistic effect, leading to an increase in intra‐tumoral Tpex cells. Potential interactions between Tpex cells and mregDCs in tumours were identified, indicating that minP1 served as an immunoadjuvant that promoted the effectiveness of anti‐PD‐L1 immunotherapy, resulting in increased Tpex cells in tumours.^47^ Thus, the utilization of the HMGN1 peptide‐based approach could potentially improve the therapeutic efficacy of ICB by expanding intra‐tumoral Tpex cells.

Despite aggressive multimodal therapy, glioblastoma remains a fatal disease. Khan and colleagues investigated the efficacy of PD‐1 ICB, a CD40 agonist, and a combination of both in a CT2A glioblastoma mouse model that contained dysfunctional CD4^+^ T cells and was unresponsive to anti‐PD‐1 ICB.^48^ The results demonstrated that a combinatorial therapy of CD40 agonist and anti‐PD‐1 ICB was found to improve survival in CT2A tumour‐bearing mice, indicating that dysfunctional CD4^+^ T cells were correlated with Tex‐term and CD4^+^ T cells played a role in the efficacy of anti‐PD‐1 ICB by controlling the severity of exhaustion. This was significant as CD4 lymphopenia is frequently found in patients with glioblastoma and might be a basis for resistance to PD‐1 inhibitors. CD40 agonism might overcome a dysfunctional CD4 compartment, leading to the expansion of Tpex and improving the efficacy of PD‐1 blockade therapy.^48^ Thus, this supports a novel synergistic immunotherapeutic approach that could be beneficial in the treatment of glioblastoma.

The successful use of cyclophosphamide‐based haploidentical stem cell transplants in clinical settings suggests that the drug may have the ability to re‐orchestrate the immune system. In various models of triple‐negative breast cancer with different intra‐tumoral immune contextures, a combinatorial therapy of intermittent cyclophosphamide, ICI, and vinorelbine has been demonstrated to activate APCs and prevent local and metastatic tumour growth in a T‐cell‐dependent manner.^49^ In addition to this, the therapy also resulted in an increase in intra‐tumoral Tpex cells and a shift in the balance between Tex‐term cells and Tpex cells, with a clear preference for the latter.^49^ Thus, the research showed that combinatorial therapy on mouse models of breast cancer can increase ICI by activating APCs and enhancing intra‐tumoral Tpex cells.

## 5 CHALLENGES AND PROSPECTS

Tpex cells are capable of recognizing and eliminating cancer cells with high specificity and potency, rendering them an ideal candidate for targeted therapy against cancer. However, there are still several challenges to overcome in the development of Tpex cell immunotherapy, including ensuring safety, efficacy, and scalability, and making it accessible to all cancer patients.

Identifying appropriate targets is crucial, as they must be expressed on cancer cells but not on healthy cells to avoid off‐target effects and harm to healthy tissues. Overcoming the immunosuppressive microenvironments created by tumours is another major challenge, as these environments can hinder the effectiveness of Tpex cell‐based therapies. Researchers are working on strategies to enhance immunity to cancer cells. Avoiding unwanted side effects, such as cytokine release syndrome, which can damage healthy tissues and organs, is also essential. Efforts are underway to reduce the incidence of these adverse effects.

Ensuring long‐term efficacy requires Tpex cells that can proliferate and persist in the body to provide ongoing protection against tumour recurrence. Researchers are exploring various methods to enhance the persistence and durability of Tpex cells. A specific subset of memory T cells, designated as resident memory T (TRM) cells, remain permanently in the tissue without recirculating through the blood.^50^ These cells are mainly found in the TLS of tumours. This localization supports their role in orchestrating local antitumour immune responses. They express markers like CD69 and CD103, which help them adhere to tissue and provide immediate immune protection in situ. In cancer, Tpex cells can differentiate into Tex‐term but retain the potential to differentiate into TRM‐like cells under certain conditions.,^51^ suggesting a degree of plasticity. Tpex and TRM cells share some transcriptional regulators, such as TCF‐1, which influence their development and maintenance.^52^ Tpex cells contribute to a sustained immune response in cancer, while TRM cells provide rapid and localized immunity in tissues. Targeting pathways that promote the differentiation of Tpex into TRM‐like cells could enhance tissue‐specific immunity and improve outcomes in cancer.

In addition, validated biomarkers that can predict clinical responses to Tpex cell‐based immunotherapies warrant further investigation. These biomarkers are crucial for optimizing patient selection and treatment outcomes. It will require increased collaboration among researchers, clinicians, and industry partners, along with significant investment in basic and translational research, to meet these challenges.

Despite these challenges, the potential of Tpex cell‐based immunotherapies for cancer treatment offers hope for improved outcomes for patients. As Tpex cells have the potential to recognize and eliminate a variety of cancer cells, there is increasing interest in the development of Tpex‐based immunotherapies for various malignancies. One of the most promising avenues for improving the specificity and efficacy of Tpex cells is through genetic engineering, employing techniques such as CRISPR/Cas9. This approach allows for directed genetic modification, thereby enhancing the Tpex cells' targeting ability and stemness. Moreover, combining Tpex cell therapy with other immunotherapies, such as ICIs, may result in enhanced clinical benefits. Notably, the use of allogeneic Tpex cells, which can be sourced from healthy donors, could provide an off‐the‐shelf therapeutic option for cancer patients. Additionally, advances in cell manufacturing techniques may increase the feasibility and scalability of Tpex cell therapies. As the field progresses, the use of Tpex cells may expand to include other diseases where pathogenic cells need to be recognized by the immune system, such as infectious diseases. In conclusion, the prospects for Tpex cell therapy are promising, providing hope for better treatments and outcomes for patients with cancer and other diseases.

## AUTHOR CONTRIBUTIONS

Ni L. wrote the manuscript.

## CONFLICT OF INTEREST STATEMENT

The authors declare no conflict of interest.
